# Trisenox Disrupts MDM2-DAXX-HAUSP Complex and Induces Apoptosis in a Mouse Model of Acute Leukemia

**DOI:** 10.7150/jca.39996

**Published:** 2020-05-12

**Authors:** Sanjay Kumar, Paul B. Tchounwou

**Affiliations:** Cellomics and Toxicogenomics Research Laboratory, NIH/NIMHD-RCMI Center for Environmental Health, College of Science, Engineering and Technology, Jackson State University, 1400 Lynch Street, Box18750, Jackson, Mississippi, MS 39217, USA.

**Keywords:** Trisenox, p53, DAXX, HAUSP, MDM2.

## Abstract

Trisenox (TX) is successfully used for both de novo and relapsed acute promyelocytic leukemia (APL) treatment. Although TX toxicity to APL cells is mediated by oxidative stress, DNA damage, cell cycle arrest, and apoptosis, its mode of action in the transgenic mice model of APL is poorly understood. We hypothesized that TX regulates cell cycle and apoptosis in APL mice by p53 activation, DNA damage, and reduced expression of MDM2-DAXX-HAUSP complex. To test hypothesis, we treated APL mice with different doses (0, 1.25.2.5.5.0 & 7.5 mg/kg body wt) of TX and collected the liver and bone marrow cells. We applied several techniques to check the expression of PML-RARα, complex molecules, and DNA damage in APL mice bone marrow cells and liver. Our findings indicate that TX reduced the expression of PML-RARα and complex molecules, induced DNA damage and activated p53 leading to cell cycle arrest and apoptosis in APL mice liver. We found that TX promoted more promyelocytes formation with dense granules in bone marrow cells. It also transmitted the DNA damage signal through protein kinase (ATM & ATR) leading to disruption of complex and activation of p53 in APL mice liver. TX induced cell cycle arrest through activation of p53, p21, and reduced expression of cyclin D1 and cyclin dependent kinases (CDK 2, 4 & 6) in mice liver. It also caused apoptosis through upregulation of caspase 3 and Bax expression, and down-regulation of Bcl2 expression. Taken together, these molecular targets provide new insights into TX mode of action in APL mice.

## Introduction

APL, a subtype of acute myeloid leukemia (AML) that is formed inside the bone marrow through a translocation mutation between chromosome 15 and chromosome 17, affects about 1,500 people in the United States annually 1,2. It results from the formation of two fusion genes (oncogenes); promyelocytic leukemia-retinoic acid receptor alpha (PML-RARα) and RARα-PML. PML-RARα fusion transcript is involved in the pathogenesis of APL whereas RARα-PML fusion transcript is an important molecular marker for the diagnosis and monitoring of APL 2,3. TX has been used successfully for treatment of all age groups of APL patients in both induction and consolidation therapy either alone or in combination with all trans retinoic acid (ATRA) with a complete remission and high survival rate 2, 4. Recently, few TX resistant APL patients have been reported in different parts of the world with X- RARα oncogenes 5, 6.

P53, is a tumor suppressor protein 7,8, induces cell cycle arrest and apoptosis in response of DNA damage and other stresses in several cancer cells 9,10. Its expression level is kept low in normal/unstressed cells by several ubiquitin ligases (E3), predominantly mouse double minute 2(Mdm2) and their isoform Mdm4/MDMX, through proteasomal degradation and ubiquitination 11. MDM2 is an unstable protein and also a negative regulator of p53 that remains associated with DAXX, and HAUSP in the form of tertiary complex 12. This complex reduces self-ubiquitination of MDM2, maintaining MDM2 ligase activity toward p53 in normal living cells. Exposure of cells to genotoxic stress [DNA damage], oxidative stress, hypoxia, and heat shock leads to MDM2-DAXX-HAUSP complex disruption, and stimulation of MDM2 self-ubiquitination and degradation leading to accumulation of p53 7, 12-16. Scientific evidence suggests that TX stimulates p53 by DNA damage, p21 induction, cell cycle arrest at G1 phase, and apoptosis in fibroblast cells 17, human gastric cancer cells 18 and in HL-60 cells 8. It has been reported that TX inhibits cell proliferation with interaction with p21 leading to cell cycle arrest and apoptosis in human myeloma cells 19, HL-60 cells 20,21, lymphoid malignant cells 22 and NB4 cells 23. TX stimulates accumulation of death domain-associated protein (DAXX), a nuclear protein that modulates transcription of death-related genes in apoptosis 24. Berberine has been reported to cause apoptosis by interaction of DAXX, disruption of MDM2-DAXX-HAUSP complex and degradation of MDM2 in acute lymphoblastic leukemia cells 12, while doxorubicin and VP-16 kill cancer cells through disruption of DAXX-MDM2-HAUSP complex, self-ubiquitination and degradation of MDM2, which leads to accumulation of p53 12, 25. Promyelocytic leukemia (PML) gene interacts with p53 inside PML-nuclear body (NB), and is actively involved in p53 dependent pro-apoptotic events 26. Promyelocytic leukemia zinc finger-retinoic acid receptor α (PLZF-RARα), an oncogene transcriptional repressor, regulates cell proliferation in APL patients by dow-regulation of p53 and p21 proteins expression 27. Activation of PML-transformation related protein (Trp53) is necessary to control leukemia-initiating cells in mouse model of APL 28. Pseudokinase Tribble 3 (TRIB3) stimulates APL progression by inhibition of p53 mediated senescence and PML-RARα stabilization. RARα / arsenic trioxide interacts with TRIB3/ PML-RARα and eradicates APL by degradation of PML-RARα 29. In the present research, we revealed TX mode of action in a mice model of APL; involving DNA damage, stress signal transmission, reduced complex molecules expression, and activation of p53 leading to cell cycle regulation and apoptosis APL mice tissues. TX also promoted more promyelocytes formation with dense granules, and reduced PML-RARα expression in bone marrow cells. Understanding the molecular mechanism of TX action in mice model of APL would be very helpful for the design of new APL drugs.

## Material and Methods

### Chemicals and Reagents

Trisenox (arsenic trioxide) was purchased from Fisher scientific (Waltham, MA). Mitochondrial isolation kit, caspase assay kit, protease inhibitor, wright-giemsa solution and glutathione assay kit were obtained from Sigma-Aldrich (St. Louis, MO). Anti-cytochrome C, anti-Bax and anti-Bcl2 were purchased by Cell Signaling Technology (Danvers, MA). Caspase 3 activity assay kit was obtained from Abcam (Cambridge, MA). Hoechst 33342, Alexa fluor 568 and Alexa fluor 568 were purchased from Life Technologies (Grand Island, NY).

### Transgenic APL mice

Acute promyelocytic leukemia (APL) transgenic mice were purchased from The Jackson Laboratory in Bar Harbor, Maine, USA. Our APL transgenic mice strain is C57BL/6-Pml^tm1(PML/RARA)Ley^/J and stock No: 017959 having gene construct, promyelocytic leukemia-retinoic acid receptor alpha (PML- RARα) widely expressed fusion proteins and tag with Cre recombinase. APL transgenic mice were kept in the Animal Core facility following the guidelines and recommendations of Jackson State University IACUC Committee. After one month of acclimatization, they were bred to produce enough mice for experimentation. Regular genotyping was done from young pups mice tail blood and proper homozygous mice containing our desired oncogene (PML-RARα ) responsible for pathogenesis of APL were maintained 29,30. Young transgenic mice (8-12 weeks old with average weight of 20-30g) were used for this experiment. Each treatment group was made of five young mice of similar weight and age. They were treated with different doses of TX (1.25, 2.5. 5.0 and 7.5 mg/kg body wt) for 21 days by a continuous intraperitoneal injection based on previous publication 30 and our standardization procedure. After 21 days of TX treatment of transgenic mice, the liver tissue was dissected and the bone marrow cells were isolated for further experimentation.

### Bone marrow isolation from transgenic mice

Young transgenic mice (8-12 weeks old with average weight of 20-30g) were treated with different doses of TX (1.25, 2.5. 5.0 and 7.5 mg/kg body wt) for 21 days continuously. After treatment, the transgenic mice were euthanized through CO_2_ asphyxiation, and bone marrow cells were isolated from the femur and tibia as described previously 31.

### Wright staining of promyelocytes inside the bone marrow cells

We made thin smears of sterile bone marrow cells of all doses TX-treated or untreated mice samples on glass slides and promyelocytes were stained with Wright-Giemsa solution as previously described 32. In brief, air dry bone marrow cells smears were placed in Wright-Giemsa solution for about 3 minutes. After incubation, slides were dipped in phosphate buffer (pH = 7.2) for 10 minutes. Then, slides were rinsed with distilled water, air dry, and the stained promyelocytes images were taken using Arcturus Laser Capture Micro dissection (LCM) System (Grand Island, NY).

### Immunocytochemistry and confocal microscopy imaging

APL bone marrow cells (1x10^5^) were cultured in presence or absence of TX and attached on poly-L-lysine coated slides. Immunocytochemistry of attached cells was performed using Ki67 antibody (dilution, 1:100) (cat# 33-4711) or p53 antibody (cat # 9282) and PML-RARα (cat# ab43152) from Life Technology, Cell Signaling or Abcam company and imaged by confocal microscopy (Olympus Company, Center valley, PA) as previously described 2.

### Tunnel Assay

The TUNEL assay is very sensitive technique to analyze DNA damage in tissue sections. In brief, we treated transgenic APL mice with different TX doses (0,1, 2, 4, 6 and 8 mg/kg body wt) and collected the livers in RIPA buffer. Liver tissues were frozen in embedding medium( Polarstat Plus) and 5µM sections were made using Cryostar NX50 (Thermo Scientific, Waltham, MA) and DNA damage was analyzed by immunochemistry and confocal imaging using Promega DeadEnd™ Fluorometric TUNEL System Technical Bulletin (Cat# G3250) or as previously described 33. Liver sections were fixed in acetone and methanol mixture at -20 ^0^ C for 5 min and permeabilized with 0.2% triton X at 4 ^0^ C for 10 min. Slides were washed with PBS two times and equilibrated in equilibration buffer for 10 min at room temperature. 100µL rTdT incubation buffer were added on each slide and incubated at 37 ^0^ C for one hour. After incubation, the reaction was terminated by dipping slides in 2X SSC for 15 min and washed 2-3 times with PBS. The slides were stained with DAPI and mounted by anti-fade solution with coverslip and nail polish. After drying of slides, confocal imaging was performed using the Fluoview confocal microscopy system (Olympus company) 2.

### Immunoprecipitation and Western blotting

After treatment of APL cells and APL mice with different doses of TX, mice liver tissue and bone marrow cells were collected and protein lysates were prepared in RIPA buffer by sonication and centrifugation. We used 500µg protein lysate of liver tissues or APL cells of each sample and immunoprecipitation (IP) performed according to standard Thermofisher Scientific protocol and described earlier 12. For regular western blotting, an equal amount (40mg) of protein lysate from control or TX treated cells or tissues were loaded per lane on a 10% SDS-PAGE gel, transferred into a nitrocellulose membrane and analyzed by western blotting by using specific antibody as described previously 2. The band intensities were quantified using Image J software.

### Immunohistochemistry (IHC)

We collected in RIPA buffer the livers of both control and APL mice treated with different doses (1, 2, 4, 6 and 8 mg/kg body wt) of TX for 21 days continuously intraperitonially. Liver tissues were frozen in embedding medium (Polarstat Plus), and 5µM sections were made using Cryostar NX50 ( Thermo Scientific, Waltham, MA). Liver sections were fixed in acetone and methanol mixture at -20 ^0^ C for 5 min and permeabilized with 0.2% triton X at 4 ^0^ C for 10 min. They were washed three times with PBS and blocked in 5% normal goat serum containing 4% BSA for 30 min at room temperature. Blocked sections were incubated in anti-p53 (1:100) and anti-MDM2 (1:100) antibodies inside humidified chamber for four hours at room temperature. Again, the sections were washed three times with PBS and further incubated with secondary antibody [Alexa fluor 488 & 594(1:1000)] for one hour at room temperature. After incubation, the sections were washed with PBS and the images were captured under fluorescence microscope, IX73 (Olympus, Center Valley, PA) and presented as shown previously 34.

### Statistical analysis

Experiments were performed in triplicates. Data were presented as means ± SDs. Where appropriate, one-way ANOVA or student paired *t*-test was performed using SAS Software available in the Biostatistics Core Laboratory at Jackson State University. P-values less than 0.05 were considered statistically significant.

## Results

### TX stimulates more promyelocytes formation and reduces PML-RARα expression

**Figure 1:**
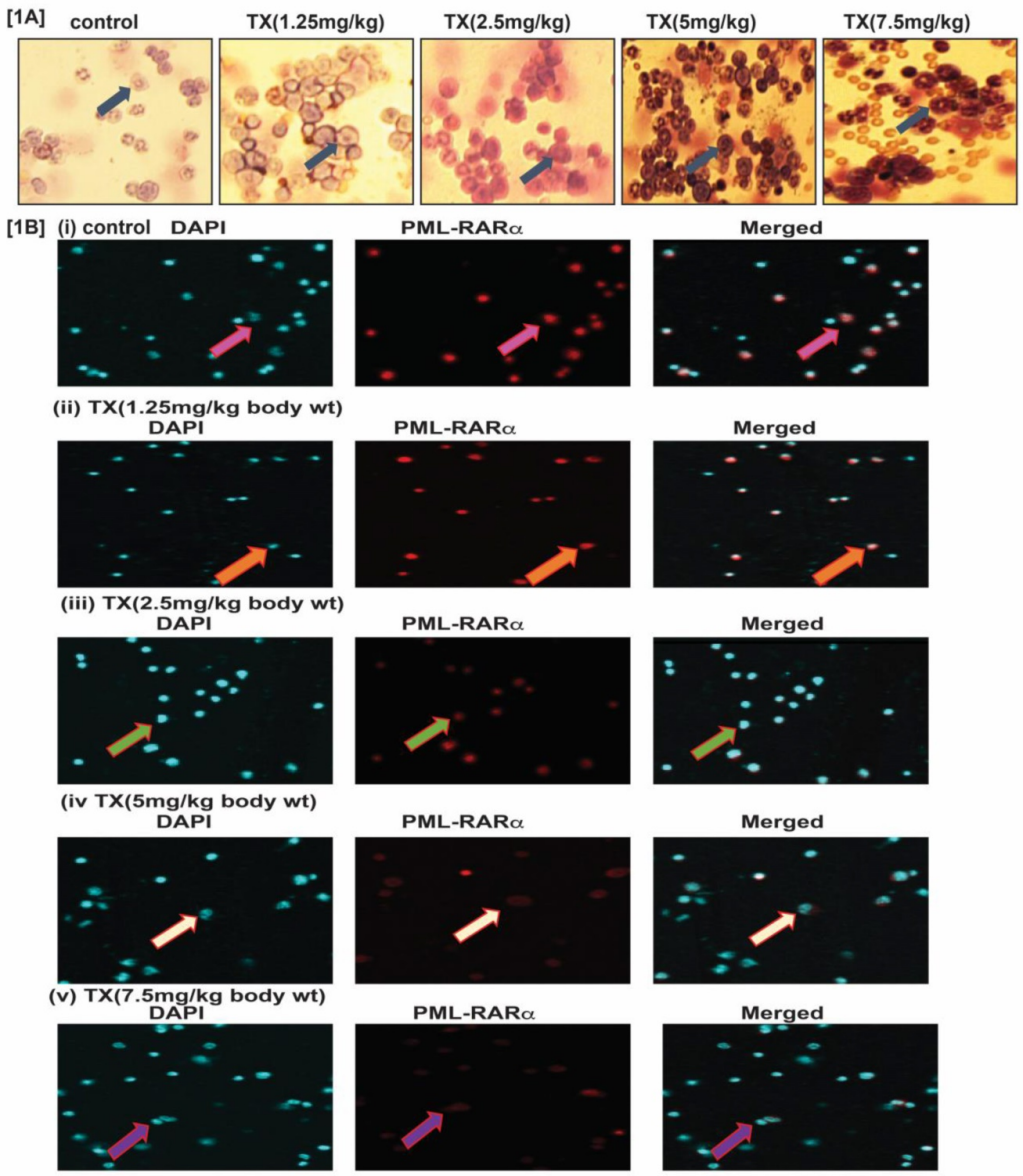
***TX stimulates more promyelocytes formation and reduces PML-RARα expression.***APL transgenic mice were treated intraperitoneally with different doses (0,1.25,2.5,5.0 & 7.5mg/kg body wt) of TX for 21 days. After treatment, the mice were sacrificed and the bone marrow cells were collected. Promyelocytes were stained with Wright staining and PML-RARα expression analyzed by immunocytochemistry and confocal imaging in APL bone marrow cells. TX promoted more promyelocytes with dense granules formation and reduced PML-RARα expression in APL mice bone marrow cells (Fig. 1A &B [i-v]).

To study TX effect on transgenic mouse model of APL, we treated mice with different doses (0, 1.25, 2.5, 5 and 7.5 mg/kg body weight) of TX and isolated bone marrow cells. We made thin smears of bone marrow cells on slides, dry, fixed with phosphate buffer, stained with Wright-Giemsa staining, and imaged by fluorescence microscopy. Our results show that TX stimulated more promyelocytes formation with dense granules in treated APL mice bone marrow cells in a dose-dependent manner (Fig.1A). Our immunocytochemistry and confocal imaging findings show that TX also reduced the expression level of PML-RARα oncogene in bone marrow cells, in a dose-dependent manner (Fig.1B[i-v]).

### TX induces DNA damage in APL mice liver

To investigate the genotoxic effect of TX on APL mice liver, we collected liver tissues and made 5µm sections after treatment with different doses of TX. We then performed immunohistochemistry and tunnel assay to assess TX-induced DNA damage in APL mice liver tissue. Our findings show that TX induced DNA damage in APL liver tissue in a dose-dependent fashion (2[i-v]).

### TX disrupts MDM2-DAXX-HAUSP complex

**Figure 3:**
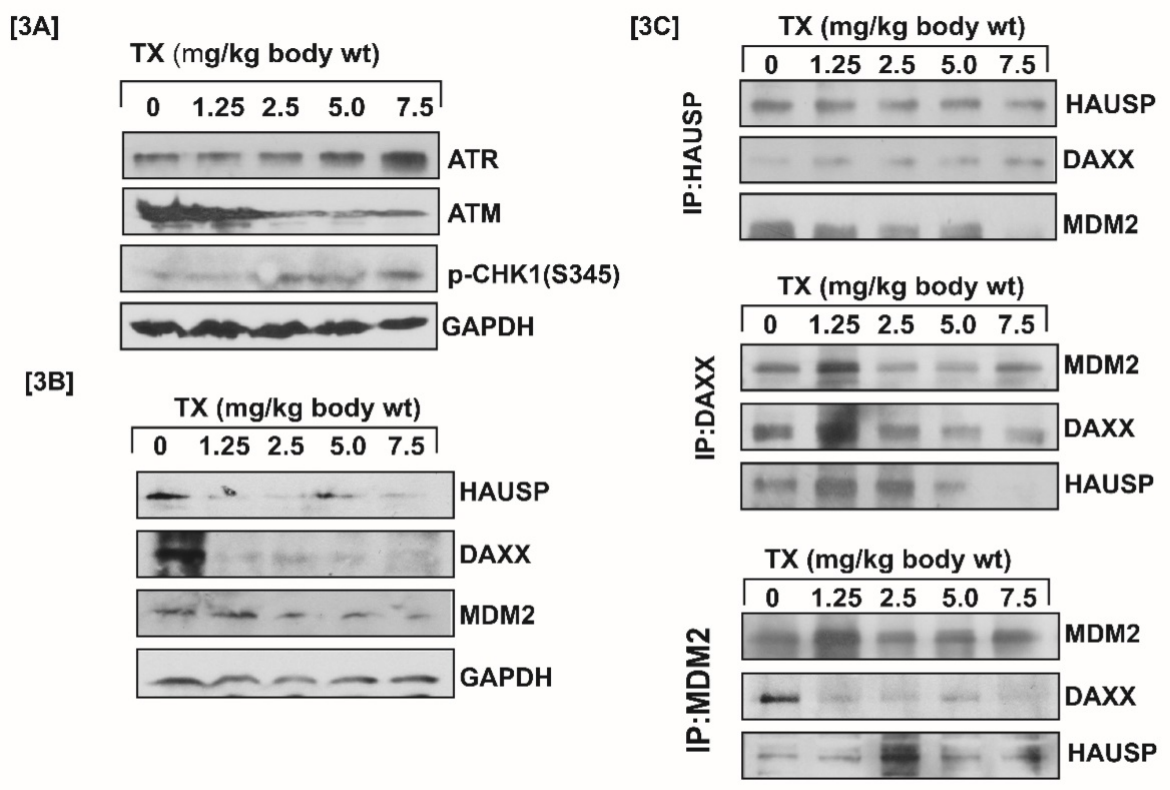
** TX disrupts MDM2-DAXX-HAUSP complex.** Both control and TX-treated APL transgenic mice were sacrificed and the liver tissues were collected in RIPA buffer. Protein lysates were made from all samples, and Western blotting was performed to assess the expression of complex molecules, protein kinases, and phosphorylation of CHK1 at Ser 345 residue using both specific and phosphoactive antibodies. TX reduced the expression of complex molecules and ATM, and stimulated the expression of ATR through phosphorylation of CHK1 at Ser 345 residue in APL liver tissues (Fig.3A&B). Immunoprecipitation (IP) was done using similar protein lysates of APL mice liver. It was found that TX also changed the association of complex molecules (Fig.3C).

Generally, DNA damage signal is transmitted by protein kinase [ATM & ATR] and its downstream CHK1& CHK2 residues phosphorylation leading to MDM2-DAXX-HAUSP complex disruption 12,15. Our results showed that TX-induced DNA damage signal was transmitted through increased ATR and reduced ATM expression with stimulation of phosphorylation of CHK1 at serine residue (Fig.3A). It also disrupted MDM2-DAXX-HAUSP complex through reduced expression and changing association of complex molecules (Fig.3B&C) in APL mice liver tissues.

### TX activates p53 in APL mice bone marrow cells

**Figure 4:**
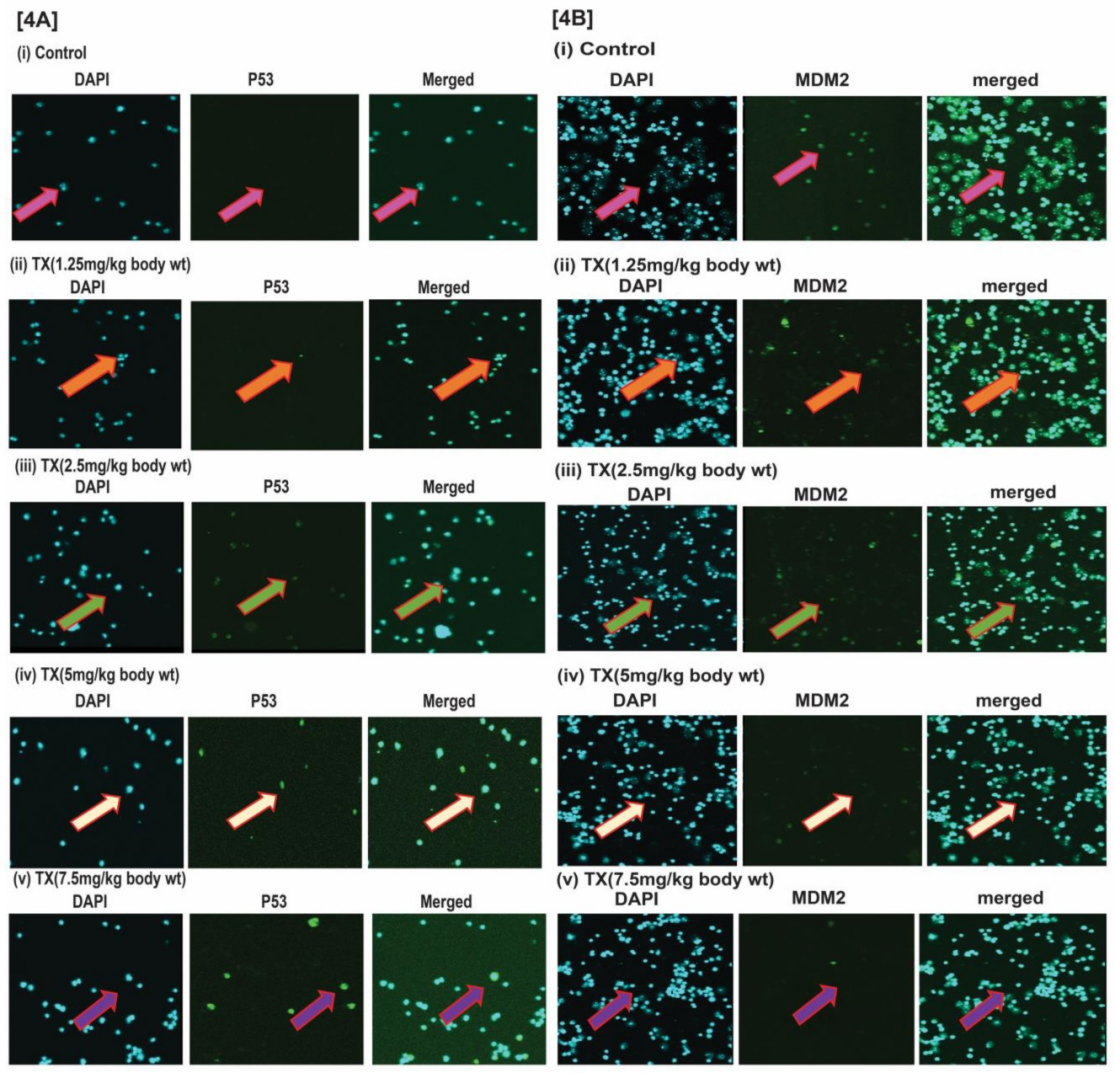
** TX activates p53 in APL mice bone marrow cells.** Immunocytochemistry was performed on both control and TX-treated APL transgenic mice bone marrow cells to check the expression levels of p53 and MDM2. TX stimulated p53 expression and reduced MDM2 expression in APL mice bone marrow cells (Fig.4A&B).

It has been reported that DNA damage disrupts MDM2-DAXX-HAUSP complex leading to activation of p53 and MDM2 degradation in many cancer cells 12, 15, 35. Our findings reveal that TX reduced MDM2 expression in a dose dependent fashion (Fig.4A&B[i-v]), leading to p53 activation in APL mice bone marrow cells.

### TX activates p53 in APL mice liver tissues

**Figure 5:**
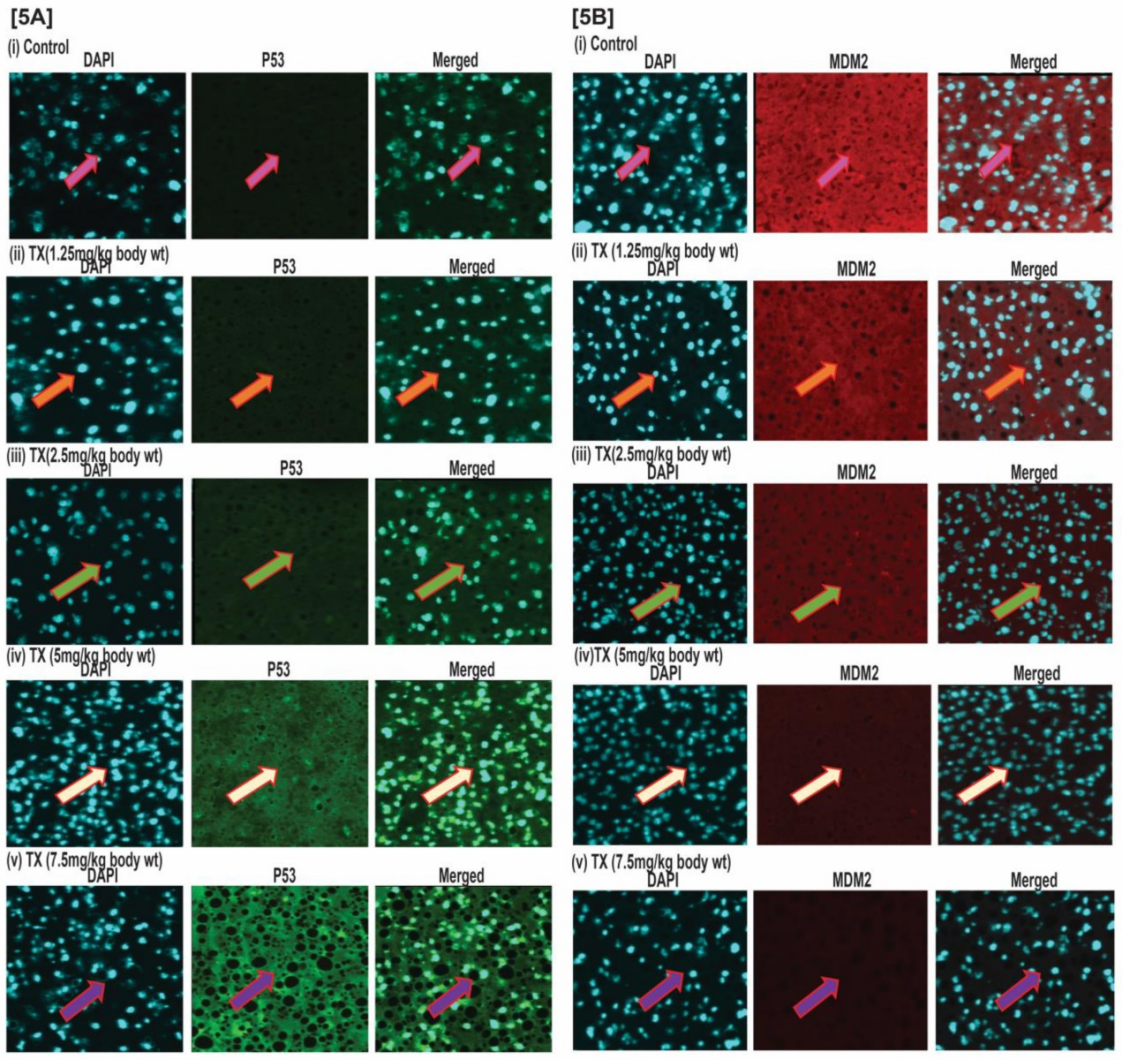
** TX activates p53 in APL mice liver tissues.** Immunohistochemistry (IHC) was performed on both control and TX-treated APL transgenic mice liver sections to check the expression levels of p53 and MDM2. TX activated p53 expression and reduced MDM2 expression in APL mice liver tissues sections (Fig.5A&B).

The results of our immunohistochemistry evaluation of APL mice liver tissue also show that TX down-regulated MDM2 expression in a dose dependent manner (Fig.5A&B[i-v]), leading to p53 activation in APL mice bone marrow cells.

### TX modulates cell cycle regulation and apoptosis

**Figure 6:**
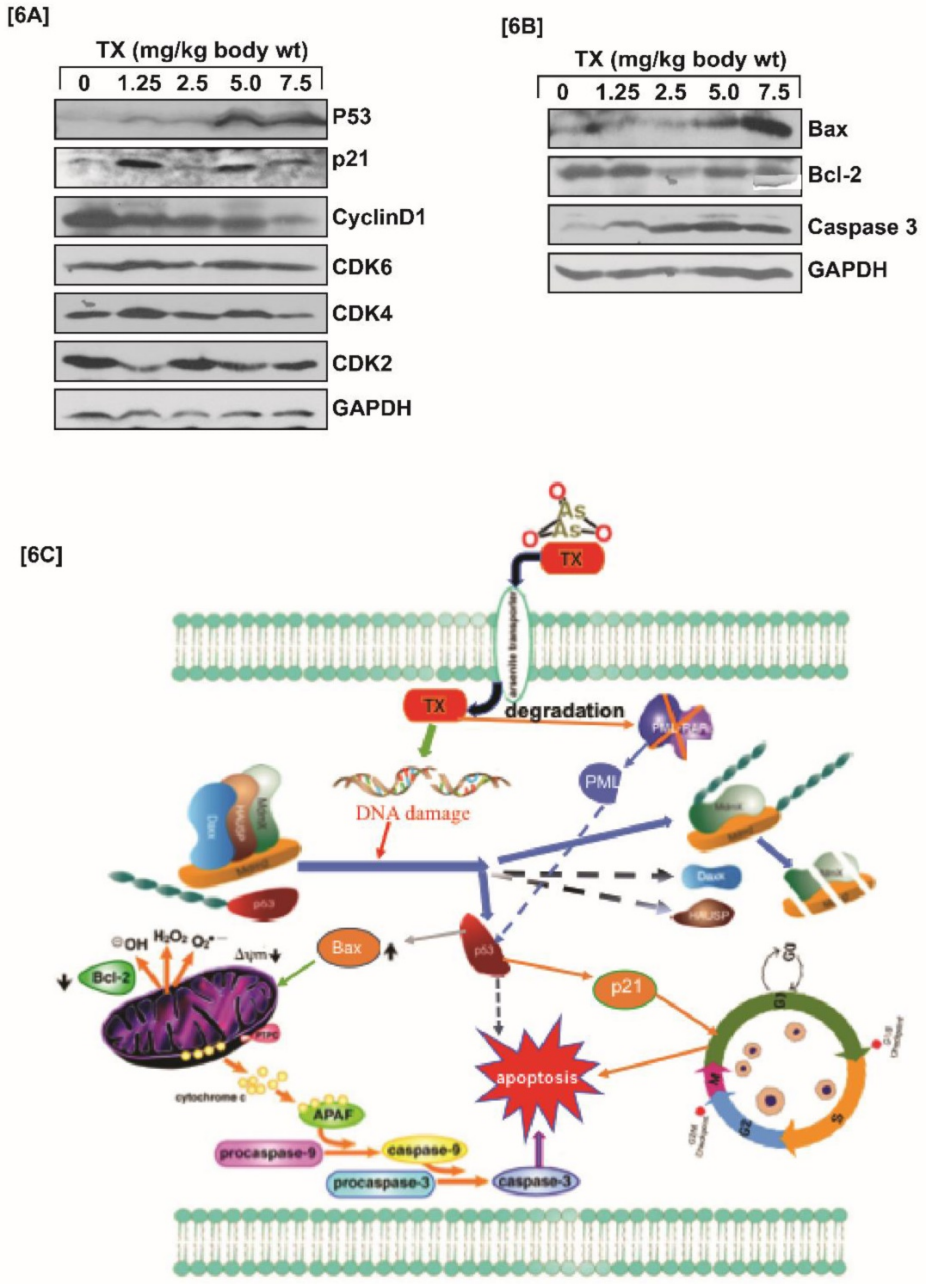
** TX involves in cell cycle regulation and apoptosis.** Both control and TX-treated APL transgenic mice were sacrificed and the liver tissues were collected in RIPA buffer. Protein lysates were made from all samples and Western blotting was performed to assess the expression levels of p53, p21, cyclin D1, CDK2, CDK4, CDK6, Bax, Bcl-2, and Caspase 3 using specific antibodies. TX significantly modulated cell cycle in APL mice liver tissues through activation of p53 and p21, and reduction of cyclin D1, CDK2, CDK4, CDK6 expression (Fig.6A). TX also induced apoptosis in APL transgenic mice liver tissue through stimulation of caspase 3 and Bax, and down-regulation of Bcl-2 expression (Fig.6B). In summary, TX-induced DNA damage signal was transmitted by protein kinases [ ATM & ATR] leading to disruption of complex molecules and down-regulation of their expression. This process also led to the activation of p53 expression, cell cycle regulation, and apoptosis in APL tissues. TX also promoted more formation of promyelocytes with dense granules in APL mice bone marrow cells (Fig.6C).

P53 is widely involved in cell cycle regulation and apoptosis in several cancers. We found that TX-induced p53 expression modulated cell cycle regulation through stimulation of p21, and reduction of cyclinD1, CDK2, CDK4, and CDK6 expression in APL mice liver tissues (Fig.6A). It also caused apoptosis in APL mice liver tissues through stimulation of caspase 3, and bax expression, and reduction of bcl-2 expression (Fig.6B).

## Discussion

Trisenox (TX) has been used successfully for the treatment of all age groups of APL patients, either alone or in combination with ATRA; leading to a maximum efficacy and high survival rate. However, TX resistance has recently been reported in few APL patients 5,6; underscoring the importance of searching for new targets of its action that may help to design new anti-leukemic drugs to cure APL patients more quickly and prevent growing resistance cases. Generally, TX enters into APL cells through diffusion and acts by various mechanisms in different pathways 17, 21,36,37. However, TX mode of action in mouse model of APL mostly remains elusive. We investigated a new mode of action of TX using a mouse model of APL. It has been reported that APL pathogenesis is caused by the formation of oncogene, PML-RARα 28,30. We found that PML-RARα was expressed in our mice model of APL and TX reduced its expression in a dose-dependent fashion (Fig.1B[i-v]). TX also stimulated the formation of more promyelocytes with dense granules in APL mice bone marrow cells (Fig.1A). Previous studies have reported that TX induces DNA damage in APL cell lines 20,21. Similarly, our finding also shows that TX caused genotoxicity in mice APL liver tissue(2B[i-v]). Accumulating evidence suggests that the DNA damage signal is transmitted by protein kinase (ATM& ATR) and its residues phosphorylation leading to disruption of MDM2-DAXX-HAUSP complex and activation of p53 12,15,35. Our findings show that TX-induced genotoxic damage was transmitted by reduced ATM and stimulated ATR expression along with phosphorylation of CHK1 residue at ser 345 residue in mice APL liver tissue (Fig.3A). It also reduced the expression of MDM2-DAXX-HAUSP complex molecules (Fig.3B) and disrupted their association (Fig.3C) in APL mice liver tissue. TX-induced disruption of complex molecules led to activation of p53 and degradation of MDM2 in APL mice bone marrow cells (Fig.4A&B[i-v]) as well as liver tissue (Fig.5A&B[i-v]) in a dose-dependent fashion. It has been also reported that p53 is widely involved in cell cycle arrest and apoptosis in several cancers 9,10. Our data show that TX-induced p53 expression was modulated cell cycle through activation of p21 and reduced expression of cyclin D1, CDK2, CDK4, and CDK6 in APL mice liver tissue (Fig.6A). TX also induced apoptosis through activation of caspase 3 and bax by reduced expression of bcl-2(Fig.6B).

In most of human cancers, p53 is mutated or remains functionally inactive by MDM2 and MDMX through E3 ligase activity. MDM2 is often involved in auto-degradation and proteasomal degradation inside cancer cells. Hence, it is always associated with DAXX and HAUSP to form MDM2-DAXX-HAUSP complex 15. Reactivation of p53 through disruption of complex and degradation of MDM2 could be an attractive and effective strategy for cancer therapy 38. The anti-cancer drugs, doxorubicin and VP-16 act inside cancer cells by successfully disrupting the MDM2-DAXX-HAUSP complex and degrading MDM2 expression 12. Trisenox inhibits the proliferation of APL cell lines through complex disruption, and MDM2 degradation leading to activation of p53. TX-induced p53 activation is involved in APL cell cycle modulation and apoptosis 35. TX-induced DNA damage signal transmission through protein kinase (ATM &ATR) led to the disruption of complex molecules and change in their association, p53 activation, cell cycle regulation, and forcing APL mice liver and bone marrow cells to undergo apoptosis.

Inside APL cells, TX exerts its pharmacological effects through different molecular mechanisms that include reactive oxygen species [ROS] induction, oxidative stress, DNA damage, and p53 activation leading to cell cycle modulation and apoptosis [8,17,21, 39-41]. P53, a tumor suppressor protein is activated in response to genotoxic and other stress-related factors, and is involved in cell cycle regulation and apoptosis of cancer cells (9.10). Generally, p53 expression is down-regulated in most cancer cells, predominantly through MDM2 and MDM4/MDMX by ubiquitination and proteasomal degradation. MDM2, a negative regulator of p53 normally associated with DAXX and HAUSP to form MDM2-DAXX-HAUSP complex [12]. The reactivation of p53 with anticancer drugs would be a promising strategy to cure cancer [38] by induction of cellular stress, MDM2 degradation, and self-ubiquitination [12,15].

It has been reported that ATO stimulates expression of p53 in resistant hepatocellular carcinoma cells 42. TX induces apoptosis in multiple myeloma and B-cell chronic lymphocytic leukemia cels through a p53-dependent signaling pathway 43,44. Accumulating evidence reveals that TX prevents growth through accumulation of p53, p21, cell cycle arrest, and apoptosis in fibroblast cells, human gastric cancer cells, myeloma cells, lymphoid malignant cells, and acute promyelocytic leukemia (APL) cell lines. MDM2, a prominent antagonist of p53 regulates its expression in cancer cells and remains associated with DAXX and HAUSP to form MDM2-DAXX-HAUSP complex. This complex would be an important target of many anticancer drugs because its disruption leads to the activation of p53 17-22. Promyelocytic leukemia (PML) gene is associated with p53 and is involved in pro-apoptotic events [26]. Promyelocytic leukemia zinc finger-retinoic acid receptor α (PLZF-RARα) stimulates cell proliferation in APL patients through a simultaneous down-regulation of the expression of both p53 and p21 proteins [27]. PML-transformation related protein (Trp53) is essential for the control of leukemia - initiating cells in mouse model of APL [28]. Pseudokinase Tribble 3 (TRIB3) promotes APL progression by inhibition of p53-mediated senescence. TX interacts with TRIB3/ PML-RARα and eradicates APL [29]. Our laboratory has previously found that TX disrupted MDM2-DAXX-HAUSP complex, reduced expression of MDM2, and activated p53 in APL cell lines [35]. However, there is no previous report evaluating it effect on MDM2-DAXX-HAUSP complex in a mouse model of APL. Hence, our findings are novel and correlate well with the current knowledge on the role of TX on the stimulation of p53 in cancer cells. For the first time, we hereby report that MDM2-DAXX-HAUSP complex is present in both liver tissues and bone marrow cells of transgenic APL mice. Most importantly, TX treatment down-regulates the expression of complex molecules and activates p53 as demonstrated in both western blotting and immunohistochemistry experiments simultaneously. This new mode of TX action in mice model of APL constitutes a novel target that may be very useful in designing new therapeutic strategies to overcome drug resistance in APL patients.

## Figures and Tables

**Figure 2:**
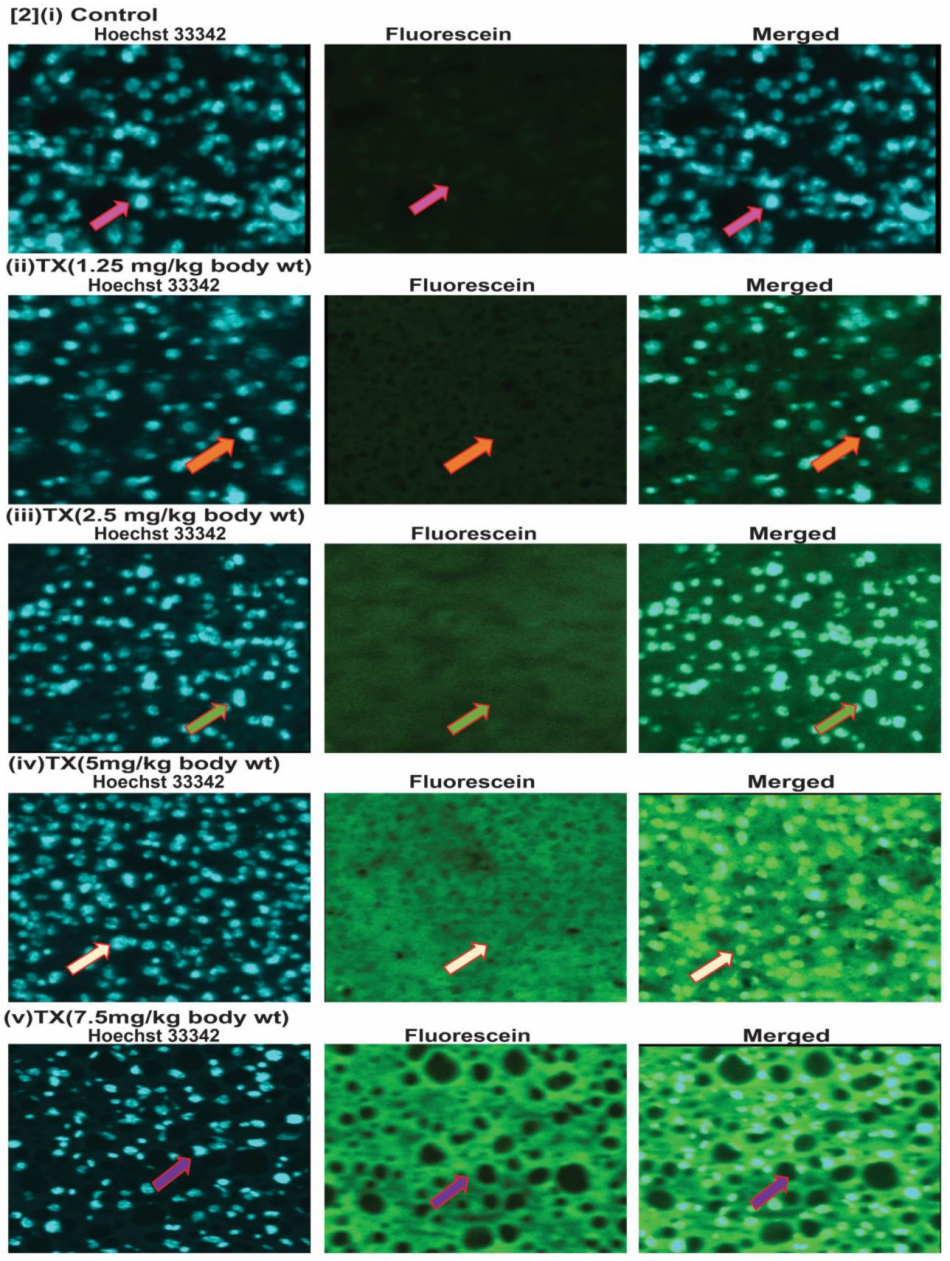
***TX induces DNA damage in APL mice liver.***Both control and TX-treated APL transgenic mice were sacrificed and the liver tissues were collected. The tissues were sectioned and sections fixed, and permeabilized, and DNA damage was analyzed by immunohistochemistry, tunnel assay, and fluorescence imaging. TX stimulated DNA damage in APL transgenic mice liver tissues (Fig.2[i-v]).
